# Comparison of ambient solvent extraction methods for the analysis of fatty acids in non-starch lipids of flour and starch

**DOI:** 10.1002/jsfa.6449

**Published:** 2013-11-18

**Authors:** Niloufar Bahrami, Lina Yonekura, Robert Linforth, Margarida Carvalho da Silva, Sandra Hill, Simon Penson, Gemma Chope, Ian Denis Fisk

**Affiliations:** a Division of Food Sciences, University of NottinghamSutton Bonington, Loughborough, LE12 5RD, UK; b Campden BRI GroupChipping, Campden, GL55 6LD, USA

**Keywords:** flour, starch, lipid, solvent extraction, neutral lipids, glycolipids, phospholipids, fatty acid

## Abstract

**BACKGROUND:**

Lipids are minor components of flours, but are major determinants of baking properties and end-product quality. To the best of our knowledge, there is no single solvent system currently known that efficiently extracts all non-starch lipids from all flours without the risk of chemical, mechanical or thermal damage. This paper compares nine ambient solvent systems (monophasic and biphasic) with varying polarities: Bligh and Dyer (BD); modified Bligh and Dyer using HCl (BDHCL); modified BD using NaCl (BDNaCl); methanol–chloroform–hexane (3:2:1, v/v); Hara and Radin (hexane–isopropanol, 3:2, v/v); water-saturated *n*-butanol; chloroform; methanol and hexane for their ability to extract total non-starch lipids (separated by lipid classes) from wheat flour (*Triticum aestivum L*.). Seven ambient extraction protocols were further compared for their ability to extract total non-starch lipids from three alternative samples: barley flour (*Hordeum vulgare* L.), maize starch (*Zea mays L*.) and tapioca starch (*Manihot esculenta* Crantz).

**RESULTS:**

For wheat flour the original BD method and those containing HCl or NaCl tended to extract the maximum lipid and a significant correlation between lipid extraction yield (especially the glycolipids and phospholipids) and the polarity of the solvent was observed. For the wider range of samples BD and BD HCl repeatedly offered the maximum extraction yield and using pooled standardized (by sample) data from all flours, total non-starch lipid extraction yield was positively correlated with solvent polarity (*r* = 0.5682, *P* < 0.05) and water ratio in the solvent mixture (*r* = 0.5299, *P* < 0.05).

**CONCLUSION:**

In general, BD-based methods showed better extraction yields compared to methods without the addition of water and, most interestingly, there was much greater method dependence of lipid yields in the starches when compared to the flour samples, which is due to the differences in lipid profiles between the two sample types (flours and starches).

## INTRODUCTION

Lipids are minor components of flour but are important in the cereal industry for a variety of functional reasons; for example in baked products such as bread, the texture and volume of the final product are strongly determined by the gas bubble retention properties of the dough. Gas bubble retention is affected by the presence of surface-active compounds, which include lipids, at the gas bubble surface. Their functionality is further highlighted in previous studies,^1^,^2^ which have shown that in a model system the addition of polar lipids to defatted flour can increase the loaf volume of baked products (e.g. bread and cake); it is also known that wheat kernel hardness and endosperm texture are dependent on friabilin proteins which, in association with polar lipids, determine end baked product quality.^3^,^4^ However, it is important to note that flour lipids undergo several significant changes during storage and this can impact, both positively and negatively, the final product quality of baked goods.^1^,^2^,^5^–^9^

Although lipids comprise only 1.5–7.0% of cereal grains,^10^ they display a large structural diversity and are unequally distributed across the wheat grain; neutral lipids originate from the rough endoplasmic reticulum and are mainly present in the germ, aleurone and scutellum, while polar lipids are more abundant in the starchy endosperm.^5^ Neutral lipids typically comprise acylglycerols, free fatty acids, sterols, sterol esters, waxes and hydrophobic pigments. In the cereal grain, triacylglycerols are the main storage lipid and are contained in small stable subcellular organelles termed oil bodies,^11^,^12^ triacylglycerols can also be found to a lesser extent in the starchy endosperm.^13^ Polar lipids, in addition to a range of minor fractions, comprise glycolipids, such as mono- and digalactosyl acylglycerides, and phospholipids which are mainly present in wheat starchy endosperm in association with friabilin proteins.^3^ Wheat flour contains mainly endosperm lipids plus a small proportion of lipids from the germ depending on the milling fraction;^14^ thus the ratio of non-polar to polar lipids and neutral–glycolipid–phospholipid can be modulated by flour milling procedures,^15^ allowing for the production of flour with the desired baking functionalities. Storage and shelf life also have a direct effect on flour lipids; the most common example being the development of free fatty acids over storage by enzymatic (lipase) and oxidative lipid degradation pathways.^16^ Among the flour lipid classes, polar lipids are probably the most important effectors on baking functionality, with minor amounts producing marked improvements in the quality of low-moisture^17^ and high-moisture dough, where the amphiphilic nature of these lipids helps to stabilize air cells and increase gas retention in the dough.^18^

In flour, ‘non-starch lipids’ can be used to refer to all endosperm lipids including those present in the outer surface of starch granules,^13^ while lipids entrapped inside the starch granules are termed ‘internal starch lipids’ and can only be extracted when starch granules are mechanically broken down or when starch granules are gelatinized. The functional properties of starch are strongly determined by the presence of non-starch lipids on the granule surface,^15^ especially during gelatinization. The importance of non-starch lipids is more evident when dealing with purified starches such as maize and tapioca, where other free lipids are absent; furthermore, tapioca originates from cassava root, so it does not contain endosperm lipids.

Given the importance of non-starch lipids in determining flour's functionality and the characteristics of the final baked products, it is of great importance to have a reliable and robust method for the accurate quantification of those lipids. This method will act as the starting point for a more thorough investigation of the various factors affecting flour quality (e.g. genotype, phenotype, processing and ageing) and on the manufacture of flours with the desired characteristics. While there is some excellent work that has been carried out over the last 10 years,^19^–^22^ there is still discussion over which extraction method would ensure the most representative extraction of non-starch lipids, including free lipids and bound lipids, in a single type of extraction, without thermal or mechanical damage;^20^,^23^,^24^ this is unsurprising given the wide variation of polarity,^25^ accessibility and location of lipids in flour, and the extremely low concentration of surface lipids in cereal and tuber starches.

Compendium methods, such as those provided by the AACC^26^ for crude lipid extraction, make use of non-polar solvents under reflux (e.g. Soxhlet and Goldsfisch); however, non-polar solvents are well known for their inability to extract polar lipids, and the need for refluxing limits the solvent choice to single low-boiling-point solvents or azeotropes. There have been a number of attempts to increase the extraction yield of non-starch lipids by using solvent mixtures, especially those containing water, as it appears to help break the associations between lipids and the flour matrix.^27^ Methods developed for extraction of wet tissues^28^ have also been applied for flour lipid extraction. In general, non-polar solvents (e.g. petroleum ether or hexane) are recommended for the extraction of free lipids and other non-polar compounds from wheat flour, and polar solvents (e.g. water-saturated *n*-butanol) are recommended for the extraction of more polar bound lipids (i.e. glycolipids and phospholipids) after extraction of free lipids;^29^ polar solvents have also been used for the extraction of total non-starch lipids, including free and bound lipids.^20^ In general, the use of a two-step solvent extraction process for the extraction of total non-starch lipids could be considered time consuming and a single-step process would be most convenient for laboratory users. Unfortunately, there is no single recommended method for the extraction of all lipid classes present in flour and starch with a single extraction solvent and, therefore, the overall aim of this work was to find a fast and robust method for the measurement of flour non-starch lipids by quantification of their associated fatty acids with a single-solvent system (i.e. only one extraction run) without the use of elevated temperatures. This was to be achieved by comparing and evaluating a range of ambient solvent systems for their ability to extract non-starch lipids from two types of flour: barley (*Hordeum vulgare* L.) and wheat (*Triticum aestivum L*.); and two types of starch: maize (*Zea mays L*.) and tapioca (*Manihot esculenta* Crantz).

## MATERIAL AND METHODS

### Material

Plain wheat flour (Asda stores Ltd, Leeds, UK), barley flour (P&B Foods, Bradford, UK), maize starch (Dr Oetker Ltd, Leeds, UK) and tapioca starch (Infinity Foods, Brighton, UK) were purchased and stored prior to use at −20 °C for no longer than 2 months. Solvents and fatty acid standards were sourced from Sigma-Aldrich (Pampisford, UK). All reagents used were of analytical grade.

### Extraction procedures

The following extraction procedures were evaluated for their capacity to extract non-starch lipids at ambient temperature:

- *Bligh and Dyer (BD)*. Samples (500 mg) were extracted as follows: vortexed with 1 mL water (10 min); to form a wet slurry, addition of 3.75 mL chloroform–methanol (1:2, v/v); vortexed for 10–15 min; addition of 1.25 mL chloroform; vortexed for 1 min; addition of 1.25 mL water; vortexed for 1 min; centrifuged for 10 min (2700 × *g*, 5 °C) and the lower phase collected by aspiration. The aqueous phase was discarded as no lipids were detected and the pellet and interfacial layer were re-extracted twice by following the same procedure described above.^28^
- *Bligh and Dyer method with acid modification (BDHCl)*. Samples were extracted following the same protocol described above for BD, but using 0.02 mol L^−1^ HCl instead of water, to improve extraction yields of acidic phospholipids; this followed the method as described by Hajra.^30^
- *Bligh and Dyer method with salt modification (BDNaCl)*. Samples were extracted as per BD, although extraction water was replaced with 0.2 mol L^−1^ sodium chloride to enhance phase separation.
- *Extraction by solvents of different polarities*. Samples (500 mg) were vortexed for 10 min with the selected solvent (3 mL) and centrifuged (2700 × *g*, 5 °C) for 10 min. The organic phase was collected by aspiration, and the sample was extracted three consecutive times. The composition and Snyder polarity^31^ of extraction solvents were as follows (abbreviation and Snyder polarity are shown in parenthesis): Bligh and Dyer based methods (BD, BDHCl, BDNaCl, 5.90), methanol (5.1); chloroform (4.1); water-saturated *n*-butanol (WSB, 4.47); methanol–chloroform–hexane, 3:2:1, v/v (CMHex, 3.77); hexane–isopropanol, 3:2, v/v^32^ (Hara, 1.62); hexane (0.1). Snyder polarity is calculated based on the ratio of solvents in the mixture multiplied by the polarity of that pure solvent.

All extracts were dried in a stream of nitrogen, stored under nitrogen and suspended in 1 mL chloroform for storage at −80 °C.

### Fractionation of lipid classes

Lipid isolates were fractionated by solid-phase extraction according to the method of Ohm and Chung, used recently by Hobbard *et al.*^22^,^33^ Silica solid-phase extraction (SPE) columns (GracePure™ SPE Silica 1000 mg/6 ml, Grace Davison Discovery Sciences, Lokeren, Belgium) were conditioned with 5 mL hexane, followed by 5 mL chloroform. Lipid extract (1 mL in chloroform) was then loaded on to the SPE column and the eluting solvents were tested for the absence of lipids, to confirm total retention of lipids by the solid phase. Neutral lipids were eluted with 10 mL chloroform–acetone (4:1, v/v), then glycolipids were eluted with 15 mL acetone–methanol (9:1, v/v), and finally 10 mL methanol was added to elute phospholipids. The elution rate was adjusted to 0.7 mL min^−1^ by applying a 846.6 Pa vacuum (6.35 mmHg) using a vacuum manifold. Quality of separation was evaluated using authentic standards of different lipid classes.

### Fatty acid analysis by gas chromatography–mass spectrometry (GC-MS)

Crude and fractionated lipid extracts were evaporated to dryness under a nitrogen stream, dissolved in chloroform (1 mL) and stored at −80 °C until analysis. Lipid samples were thawed, combined with internal standard (40 µg 20 µL heptadecanoic acid–chloroform) and derivatized to fatty acid methyl esters (FAME) with 200 µL of 0.25 mol L^−1^ trimethyl sulfonium hydroxide (TMSH) in methanol (10 min at ambient temperature).^34^ FAME samples (1 µL) were then separated by gas chromatography (Carlo Erba GC 8000, Milan, Italy; ZB-FFAP column, 30 m, 0.25 mm i.d.; oven temperature 120 °C held for 1 min then ramped at 5 °C min^−1^ to 125 °C, then ramped to 260 °C at 10 °C min^−1^) and FAME were detected by mass spectrometry (DSQ II Thermoelectron, Austin, TX, USA; positive ion mode, full scan from 50 to 385 *m*/*z*, scan rate 500 amu s^−1^ and scan time of 0.69 s, source temperature 200 °C). Compound identification was achieved by matching with database mass spectra (NIST/EPA/NIH Mass Spectral Library, Version 2.0d, NIST, Gaithersburg, MD, USA), and comparing retention time and mass spectra with those of authentic FAME standards (Supelco, Bellafonte, PA, USA). Quantification was achieved using the ratio of each target compound's peak area relative to that of the internal standard.

The total lipid concentration in each sample was expressed as the sum of the five major fatty acids (palmitic, stearic, oleic, linoleic and linolenic acid) as g kg^−1^ flour or g kg^−1^ starch, to maximize comparability and minimize analytical variation that may be introduced by including minor lipid fractions. Triplicates of all samples were extracted three times by each extraction protocol (three sequential extraction steps), at ambient temperature. All results are expressed as mean ± standard deviation.

### Experimental design and statistical analysis

All experiments were conducted with a fully balanced experimental design, with randomized analysis order and three sample replicates. Comparison between extraction methods was carried out by one-way analysis of variance and Tukey's HSD *post hoc* test using IBM SPSS Statistics, version 19 (IBM Corp., Armonk, NY, USA). Probability values lower than 0.05 were considered significant. Linear correlation (Pearson's correlation coefficients) was used to identify trends between solvent polarity (Snyder polarity) and extraction yield of total fatty acids, neutral lipids, glycolipids and phospholipid from wheat flour data to identify correlations (IBM SPSS Statistics, version 19). Principal component analysis (PCA) was performed on extraction yield, fatty acid profile, solvent polarity and water ratio in the solvent mixture, with values relative to total lipid per sample standardized (value − mean of lipid extracts per sample / standard deviation of lipid extracts per sample) across the different sample matrices using STATISTICA*®* software (Statistica release 7, Statsoft Inc., Tulsa, OK, USA).

## RESULTS AND DISCUSSION

### Impact of choice of solvent on the yield of extraction of lipid classes from wheat flour

For total lipids, BD methods (including the variants using HCl and NaCl) showed the highest extraction yields (Table1), followed by methanol and WSB, although there was no statistically significant difference. The remaining extraction solvents showed lower yields compared to BD methods; hexane was the solvent system with the lowest extraction yield. Hexane extraction yields were 35% lower than the BD techniques; this is presumably due to its low polarity. For external comparison, it should be noted that total lipid values correspond to the sum of the five major fatty acids and therefore do not accommodate non-fatty acid lipid components.

**Table 1:** Yield of extraction (g kg^−1^ flour ± standard deviation) of total non-starch lipid, neutral lipids, glycolipids and phospholipids from wheat flour by different solvent systems and the proportion of each lipid class within total lipids extracted as percentages in parentheses

Solvent system	Snyder polarity	Extraction yield (g fatty acid kg^−1^ flour)			
		Total lipids	Neutral lipids	Glycolipids	Phospholipids
BD^a^	5.90	9.46 ± 0.66d	5.08 ± 0.52bc (53.6%)	2.98 ± 0.32d (31.5%)	1.40 ± 0.23bcd (15.0%)
BDHCl^a^	5.90	9.76 ± 0.19d	5.43 ± 0.16c (55.6%)	2.48 ± 0.15cd (25.4%)	1.85 ± 0.10d (19.0%)
BDNaCl^a^	5.90	9.56 ± 0.44d	5.12 ± 0.23bc (53.5%)	2.82 ± 0.32cd (29.5%)	1.62 ± 0.30cd (17.0%)
Methanol	5.10	8.53 ± 0.53bcd	4.94 ± 0.22bc (58.0%)	2.19 ± 0.28bc (25.6%)	1.39 ± 0.09bc (16.3%)
WSB	4.47	8.79 ± 0.23cd	5.13 ± 0.16bc (58.4%)	2.46 ± 0.30cd (28.0%)	1.20 ± 0.02abc (13.7%)
CMHex	3.77	7.42 ± 0.33abc	3.76 ± 0.26a (50.6%)	2.19 ± 0.16bc (29.5%)	1.47 ± 0.08cd (20.0%)
Chloroform	4.10	7.69 ± 0.72abc	4.56 ± 0.37abc (59.3%)	1.67 ± 0.20ab (21.7%)	1.46 ± 0.14cd (19.0%)
Hara	1.62	7.11 ± 0.41ab	3.89 ± 0.28a (54.6%)	2.25 ± 0.11bcd (32.0%)	0.97 ± 0.11ab (13.6%)
Hexane	0.10	6.30 ± 0.92a	4.42 ± 0.45ab (70.6%)	1.09 ± 0.37a (17.0%)	0.78 ± 0.14a (12.4%)

a For BD methods, Snyder polarity refers to the first solvent mixture, i.e. water–chloroform–methanol (1:1.25:2.5, v/v/v).

Means with different letters within a column are significantly different by ANOVA and Tukey's HSD test (*P* < 0.05).

Total lipids extracted by each solvent regime were fractionated by SPE, prior to analysis by GC-MS, into fractions enriched in neutral lipids, glycolipids and phospholipids. BDHCl extracted the maximum neutral lipids (5.43 ± 0.16 g kg^−1^ flour), although there was no significant difference between BDHCl and BDNaCl, BD, methanol and WSB. Chloroform and hexane had intermediate yields, while CMHex and Hara had the lowest extraction yields (Table1). When comparing extraction protocols for glycolipid extraction, BD showed the best performance (2.98 ± 0.32 g kg^−1^ flour), followed by BDNaCl, BDHCl, WSB and Hara (not significantly different from BD). Methanol and CMHex had intermediate performance, and chloroform and hexane had the lowest glycolipid yield of extraction with absolute yields reduced by 63% and 43% respectively when compared to BD (Table1). For phospholipids, BDHCl was the method with the highest yield (1.85 ± 0.10 g kg^−1^ flour), while hexane was the least efficient, with 58% reduction in extracted phospholipid (Table1).

The range of relative amounts of each lipid class extracted is shown in Table1 (51–71% neutral lipids, 17–32% glycolipids and 12–20% phospholipids), which highlights the differences between the various solvent systems. A selective enrichment in neutral lipids can be seen in the more non-polar solvent, hexane, which is presumably due its inability to extract the more polar lipid analytes from the matrix, or an inability to diffuse through or hydrate the matrix sufficiently to extract bound lipid fractions.^35^

**Figure 1:**
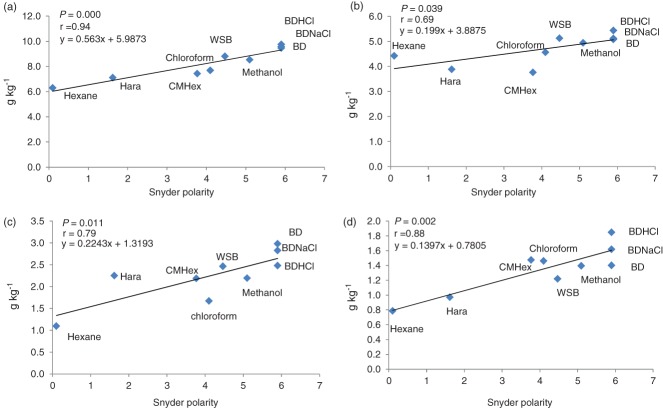
Correlation between the solvent polarity (Snyder polarity) and extraction yields (g kg^−1^) of total (a) fatty acids, (b) neutral lipids, (c) glycolipids and (d) phospholipids from wheat flour (correlations are expressed by *r* as correlation coefficient and *P*-value and are considered significant when <0.05).

Given the wide variation in ability of solvents to diffuse through the sample and differential abilities to solubilize individual lipid classes,^25^ it is not surprising that the choice of solvent for optimal extraction has been under discussion.^23^,^24^,^28^ In recent years a range of different solvent systems optimized for specific analytes have typically been used to extract non-starch lipids from wheat flour^20^ but still more studies are needed to determine a fast and efficient one-step procedure for the extraction of total lipids, including different lipid classes with different polarities.^19^ Our results confirm that different methods provide high yields for specific lipid classes, and in general high-polarity solvents allowed better extraction of total lipids (Table1). Indeed, we observed a significant correlation between solvent polarity and the yield of total lipids (*r* = 0.94, *P* = 0.000), which was driven mainly by the positive correlation between polarity and yield of phospholipid (*r* = 0.88, *P* = 0.002) and glycolipid (*r* = 0.79, *P* = 0.011); neutral lipid yields were weakly correlated with solvent polarity (*r* = 0.69, *P* = 0.039) (Fig. 1).

The method dependence of the lipid classes may be in part due to the fact that the addition of water to the solvent mixture may promote partial hydration of the flour particles and enhance solvent accessibility. In addition, some lipid species occur as complexes with carbohydrates and proteins, so extraction is more difficult and only possible when the solvent is able to overcome the hydrophobic, van der Waals and ionic forces that exist between lipid and the matrix.^36^ Historically, water-saturated *n*-butanol has been the solvent of choice for extraction of wheat lipids and preparation of lipid-free flours;^37^ however, the high boiling point of *n*-butanol makes sample preparation lengthy as the solvent usually has to be evaporated prior to derivatization and quantification of fatty acid esters or gravimetric lipid determinations.

As our aim was to find a fast and robust ambient method for quantitative measurement of flour lipids by quantification of fatty acid methyl esters, the extraction of non-lipids by a water-rich solvent would not interfere with the results. Thus, the BD methods^28^ appear to be the best choice, as they use a water-rich solvent (water–chloroform–methanol; 1:1.25:2.5, v/v/v) in the first step of extraction, and gradually lower the polarity to ensure extraction of neutral lipids. Our results corroborate previous studies that have shown BD-based methods as a good choice for extraction of lipids from wheat flour, as they offered the highest yield in all lipid classes; furthermore, BDHCl was significantly more effective than WSB for the extraction of phospholipids (>25%). Among BD methods, there was a tendency of BDHCl to offer greater extraction yields for neutral lipids (including free fatty acids) and phospholipids, although no significant differences were observed (Fig. 1). These results are in line with previous reports on the addition of acid to the aqueous phase to improve the recovery of polar (acidic) phospholipids.^30^

The relative abundance of key fatty acids in each lipid fraction is hereinafter discussed.

The five major fatty acids present in the neutral lipid fraction were palmitic (C16:0), stearic (C18:0), oleic (C18:1), linoleic (C18:2) and linolenic acid (C18:3). To increase comparability and maximize reproducibility only the five major fatty acids listed are considered here (Table2), although others were identified. The presence of minor fatty acids has been studied by many authors previously and therefore is not included for clarity. In general, linoleic acid was the most predominant neutral lipid fatty acid, followed by palmitic acid, oleic acid, stearic acid and linolenic acid. It has been shown by previous studies that the triglyceride fraction constitutes about 30–36% of the wheat flour neutral lipids^38^–^40^ and that the fatty acid composition of esterified fatty acids (e.g. glycerides) is 60–70%, 17–24%, 7–15%, 0.3–1.2% and 2.5% for linoleic, palmitic, oleic, stearic and linolenic acid, respectively. When comparing extraction methods by the abundance of individual fatty acids within the neutral lipid fraction it was found that the range of fatty acids within this lipid class was 42.0–50.8% for linoleic, 23.6–30.2% for palmitic, 16.2–20.0% for oleic, 3.36–7.33% for stearic and 1.44–2.14% for linolenic acid.

**Table 2:** Fatty acid profile within lipid classes extracted from wheat flour by different methods expressed as percentage fatty acid per lipid class

Solvent	Fatty acid				
	Palmitic	Stearic	Oleic	Linoleic	Linolenic
Neutral lipid					
BD	27.5 ± 0.9bc	6.30 ± 0.04c	16.2 ± 0.9a	48.1 ± 1.9ab	1.72 ± 0.05a
BDHCl	26.6 ± 0.1bc	6.14 ± 0.22c	16.9 ± 0.2ab	48.6 ± 0.5b	1.59 ± 0.34a
BDNaCl	27.2 ± 0.2bc	6.15 ± 0.74c	17.3 ± 0.0abc	47.5 ± 1.0ab	1.70 ± 0.16a
Methanol	26.6 ± 0.3bc	3.36 ± 0.07a	19.2 ± 0.1cd	48.9 ± 0.4b	1.79 ± 0.57a
WSB	26.6 ± 0.2bc	4.01 ± 0.13ab	17.2 ± 0.4abc	49.9 ± 1.1b	2.14 ± 0.21a
CMHex	28.9 ± 0.6cd	7.33 ± 0.16d	17.5 ± 1.4abc	44.3 ± 0.1ab	1.78 ± 0.30a
Chloroform	23.6 ± 1.5a	4.69 ± 0.16b	18.8 ± 0.9bcd	50.8 ± 2.2b	2.01 ± 0.35a
Hara	30.2 ± 1.1d	6.18 ± 0.43c	20.0 ± 1.0d	42.0 ± 3.1a	1.44 ± 0.26a
Hexane	26.6 ± 0.3ab	3.36 ± 0.07b	19.2 ± 0.1d	48.9 ± 0.4ab	1.79 ± 0.57a
Glycolipid					
BD	19.3 ± 0.3abc	1.24 ± 0.16a	13.4 ± 0.9ab	63.5 ± 2.2de	2.27 ± 0.46ab
BDHCl	20.2 ± 1.6abc	2.40 ± 0.88ab	13.2 ± 1.2a	61.7 ± 2.8cde	2.31 ± 0.07ab
BDNaCl	16.6 ± 0.4a	2.16 ± 0.06ab	11.6 ± 0.2a	67.4 ± 0.4e	2.01 ± 0.43ab
Methanol	21.1 ± 1.5bc	2.75 ± 0.14bc	14.6 ± 0.2ab	58.8 ± 1.5cd	2.53 ± 0.53b
WSB	19.4 ± 0.7abc	2.27 ± 0.02ab	13.2 ± 0.5a	62.3 ± 1.1cde	2.64 ± 0.44b
CMHex	22.1 ± 2.6bc	4.06 ± 0.47cd	15.3 ± 1.9abc	55.8 ± 1.3bc	2.26 ± 0.60ab
Chloroform	29.5 ± 1.9d	6.54 ± 0.08e	19.3 ± 3.4c	41.9 ± 3.1a	1.89 ± 0.18ab
Hara	18.3 ± 0.1ab	2.72 ± 1.18bc	12.7 ± 0.0a	64.7 ± 1.9de	1.23 ± 0.02a
Hexane	22.5 ± 0.4c	4.50 ± 0.90d	17.4 ± 0.8bc	51.6 ± 6.2b	2.88 ± 0.00b
Phospholipid					
BD	29.4 ± 1.0bc	nd	13.7 ± 2.4ab	55.4 ± 5.0a	1.47 ± 0.20ab
BDHCl	29.6 ± 1.9bc	nd	12.2 ± 0.5ab	56.2 ± 0.7ab	1.76 ± 0.22ab
BDNaCl	26.9 ± 3.2ab	nd	14.3 ± 2.4ab	55.9 ± 6.3ab	2.71 ± 1.18b
Methanol	29.5 ± 1.4bc	nd	13.3 ± 0.3ab	55.8 ± 0.1ab	1.22 ± 0.28ab
WSB	30.2 ± 0.0bc	nd	12.7 ± 0.8ab	55.3 ± 0.3a	1.61 ± 0.48ab
CMHex	27.0 ± 0.1ab	nd	12.8 ± 0.1ab	59.4 ± 1.8ab	0.56 ± 0.13a
Chloroform	31.8 ± 0.7c	nd	13.7 ± 0.9ab	52.6 ± 0.0a	1.97 ± 0.20ab
Hara	31.4 ± 0.5c	nd	15.0 ± 0.7b	51.0 ± 2.9a	1.98 ± 0.71ab
Hexane	24.3 ± 1.5a	nd	11.3 ± 0.5a	64.0 ± 1.2b	nd

‘nd’ indicates that the fatty acids were not detected. Means with different letters within each column in each lipid class are significantly different by ANOVA and Tukeys HSD test (p < 0.05).

Previous studies have shown that the principal glycolipids in wheat are monogalactosyl and digalactosyl diglycerides and that the relative fatty acids present within their structures are 79% and 73% linoleic; 5.5% and 11% palmitic; 8.2% and 7.3% oleic; 6.3% and 7.3% linolenic; and 0.6% and 1.4% stearic, respectively.^38^ The fatty acid profiles of glycolipids identified herein was reasonably similar, although, as can be expected of any natural material, some differences were observed, which could be attributed to a wide range of environmental factors. Linoleic acid was the most abundant fatty acid within the glycolipid fraction, followed by palmitic, oleic, stearic and linolenic acid (Table2). When comparing different solvent regimes for their selectivity to extract different glycolipids by their fatty acid composition it was shown that the proportions were 41.9–67.4% for linoleic, 16.6–29.5% for palmitic, 11.6–19.3% for oleic, 1.24–6.54% for stearic and 1.23–2.88% for linolenic acid. The wide method dependence of the glycolipids, as a function of solvent choice, is driven by the ability of the solvent to hydrate the sample, diffuse through the matrix and overcome the association of the bound glycolipids with cellular structures.

### Comparison of the ability of seven ambient solvent systems to extract non-starch lipids from flours and isolated starches

Lipids in starch-rich fractions of flour generally contain more bound lipids and less free lipids than flour lipids, and it was hypothesized that their extraction ability would therefore be more dependent on the solvent extraction conditions chosen; we therefore compared BD-based methods and a range of selected solvents for their ability to extract lipids from two flour sources (barley and wheat) and two starch sources (maize and tapioca). Fatty acid profile of each sample is shown in the supporting information.

Barley and wheat flour contain total fatty acids at concentrations of 25 ± 2.1 and 11 ± 2.5 g kg^−1^ flour, respectively; maize and tapioca starches had lower fatty acid concentrations of 0.77 ± 0.10 and 0.22 ± 0.05 g kg^−1^ starch, respectively. The measured total lipids are comparable to those presented in the literature, with barley and wheat having published values of 20–70^41^–^44^ and 13 g kg^−1^, 45 respectively, and 0.5 and 0.8 g kg^−1^ for maize and tapioca, respectively.^46^

**Figure 2:**
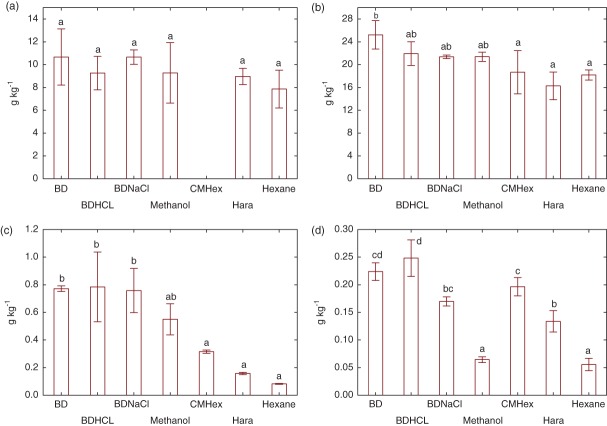
Total fatty acid (g kg^−1^) extracted from (a) wheat flour, (b) barley flour, (c) maize starch and (d) tapioca starch. Means with different letters within each figures are significantly different (ANOVA and Tukey's HSD test, *P* < 0.05, *n* = 3). CMHex for wheat was excluded due to sample loss.

When comparing extraction regimes applied to barley flour, BD extracted the maximum total fatty acids, although there was no statistically significant difference between this technique, the acid and salt-modified Bligh and Dyer methods (BDHCl and BDNaCl) and the direct methanol extraction. CMHex, Hara and hexane were significantly less efficient than BD (Fig. 2).

Among the starches, BDHCl and BD showed the maximum extraction yields (Fig. 2). In the tapioca starch, BDNaCl resulted in a statistically lower yield compared to BDHCl. Hexane gave the lowest extraction yield, while the other methods (CMHex, Hara and methanol) offered variable results depending on the analysed sample (Fig. 2). The method dependence was more significant in the starch samples compared to the flour samples and there was a significant difference between the tapioica and maize samples.

The method dependence of the analytes discussed is clearly shown in Fig. 2 and highlights the risk of assuming that one method will be effective across all similar matrices and derived isolates (e.g. starch derived from flour). The dependence of extraction yield on both sample and analyte is further discussed below, using principal component analysis (PCA) to illustrate the factors influencing the performance of the different methods for lipid extraction applied to flours and starches.

**Figure 3:**
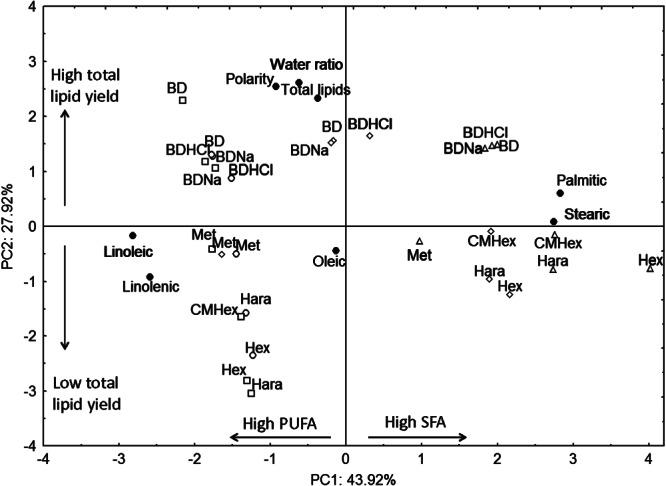
Principal component scores for lipid extraction methods applied to wheat (open circle) and barley (open square) flour, and maize (open diamond) and tapioca (open triangle) starches. Principal component loadings for variables (extraction yields) are shown as filled circles, methanol and BDNaCl extraction methods are abbreviated to Met and BDNa for clarity and other abbreviations are as given in the Experimental section.

Using pooled standardized data from all samples (normalized within each sample type by total lipid to explain differences in extraction methodology), total lipid extraction yields were positively correlated (Pearson's correlation) with solvent polarity (*r* = 0.5682, *P* < 0.05) and also with the water ratio in the solvent mixture (*r* = 0.5299, *P* < 0.05). When the data are represented by PCA (Fig. 3), PC1 and PC2 accounted for 71.84% of the variability produced by the different extraction methods. PC1 loadings were mainly related to the fatty acid profile and was positively correlated with the saturated fatty acids palmitic (*r* = 0.9432, *P* < 0.05) and stearic (*r* = 0.9149, *P* < 0.05), and negatively correlated with the polyunsaturated fatty acids linoleic (*r* = 0.9401, *P* < 0.05) and linolenic (*r* = 0.8649, *P* < 0.05). PC2 loadings were mainly driven (all positively correlated) by solvent polarity (*r* = 0.8479, *P* < 0.05), water ratio in the solvent mixture (*r* = 0.8690, *P* < 0.05) and the total lipid extraction yield (*r* = 0.7752, *P* < 0.05). Wheat and barley flour samples were clustered along the PC2 axis, confirming a positive correlation between solvent polarity, water ratio and total lipid yields in these two flour types. In addition, for wheat and barley flour samples the extraction methods were not distributed along PC1, indicating no major shift in the fatty acid profile regardless of the method used and relatively similar fatty acid profiles in wheat and barley flours. On the other hand, for the starch samples, extraction methods are distributed along PC1 (fatty acid profile), indicating a wider variation of fatty acid profiles across the different extraction methods; this is confirmed in Fig. 2. The BD method and its variants (HCl and NaCl) generally showed more efficient extraction compared to methods using solvents without addition of water, as denoted by the positioning of all BD, BDNaCl and BDHCl on the positive side of PC2, as opposed to all other extraction methods in the negative side. It was expected that starch samples would contain a greater proportion of polar lipids than flour samples and that the greater differences in fatty acid profile (across the different methods) in the starch samples may be explained by the presence of polar lipids interacting with the starch granule surface; water in the BD-based techniques is proposed to improve the solvent extraction efficiency by facilitating the dissociation of lipids from other components^27^ (e.g. starch and protein); modification of BD using NaCl shows a trend towards a reduced extraction of total lipids, which is more evident in the starch samples and may be explained by the formation of fatty acid salts and subsequent increase in solubility of the fatty acids in water,^17^ which would reduce their solubility in the organic phase. On the other hand, modification of BD using HCl in starch samples improved total extraction yield and this could be explained through the presented work, as BD using HCl showed a tendency towards greater recovery of phospholipids (Table1).

Solvents that are used to extract total lipids must demonstrate a high solubility for a wide range of lipidic compounds and be sufficiently polar to remove the lipids from their associations with non-lipid compounds such as carbohydrates, proteins and cell membrane proteins,^36^ but not excessively polar such that the solvent cannot dissolve non-polar lipids.^47^ It is therefore virtually impossible to have a single monophasic solvent that is suitable for low-temperature extraction for all lipid groups and to provide access through the matrix. The use of a ternary solvent (BD or BDHCl) that has two extraction stages with decreasing polarity has been shown here to be the most efficient and robust technique for extraction of lipids from flour and starch fractions. The BD extraction protocol and that of its associated variants (BDHCl) are probably most efficient due to the approach of the protocol: an initial monophasic ternary solvent extraction that disrupts lipid–cell associations and solubilizes polar lipids, followed by a biphasic system whereby non-lipids are partitioned out of the extraction phase. It should be noted that QUASIMEME,^48^ a multi-laboratory trial, showed that small differences in the BD method can result in significant differences in inter- and intra-laboratory results, and therefore methodologies should be followed strictly to ensure comparability and optimized extraction efficiencies; and that the BD method does result in the formation of interfacial material that needs to be treated carefully and could ultimately result in greater analytical variation over time.

Future development work is expected to include a more rigorous investigation of the impact of hydration state (i.e. moisture content) of the analyte matrix and the application of a wider range of solvents on specific industrial milling fractions to evaluate the impact of de-branning and flour milling on specific solvent efficiencies, especially with respect to the mechanical damage of starch granules through processing.

## CONCLUSION

Bligh and Dyer based methods (BD, BDNaCl and BDHCl) gave better extraction yields compared to methods without the addition of water, and there was greatest method dependence in the starches when compared to the flour samples, which we believe is important for the academic community to recognize and to incorporate into their work in future studies.
