# Supplementary material for: Comparison of ambient solvent extraction methods for the analysis of fatty acids in non-starch lipids of flour and starch
Source: J Sci Food Agric. 2013 Nov 18;94(3):415–23. doi: 10.1002/jsfa.6449 (PMC4283047; doi:10.1002/jsfa.6449)
Supplement: Appendix S1 — Supplementary Data: [file jsfa0094-0415-SD1.doc]

Supplementary Data:

Fatty acid profile extracted from wheat flour, barley flour, maize starch and tapioca starch by different methods expressed as percentage of total fatty acids (nd indicates that the fatty acids were not detected).

|  | Fatty acids (% of total fatty acids) | | | | |
| --- | --- | --- | --- | --- | --- |
|  | Barley flour | | | | |
|  | palmitic | stearic | oleic | linoleic | linolenic |
| DB | 21.1±0.7 | 2.08±0.02 | 20.0±0.0 | 53.5±0.9 | 3.12±0.09 |
| BDHCl | 22.7±1.3 | 2.54±0.17 | 20.4±0.1 | 51.2±1.1 | 3.03±0.13 |
| BDNaCl | 23.6±0.1 | 2.07±0.29 | 18.9±0.4 | 52.6±0.9 | 2.66±0.32 |
| CMHex | 23.3±2.7 | 2.48±0.28 | 19.3±2.1 | 51.4±1.0 | 3.24±0.06 |
| Hara | 21.2±0.5 | 2.37±0.16 | 20.2±0.3 | 52.8±0.3 | 3.27±0.01 |
| Methanol | 21.7±0.0 | 1.86±0.02 | 20.1±0.1 | 52.9±0.2 | 3.36±0.05 |
| Hexane | 21.0±0.1 | 1.89±0.05 | 21.5±0.1 | 52.3±0.1 | 3.18±0.03 |
|  |  |  |  |  |  |
|  | Wheat flour | | | | |
|  | palmitic | stearic | oleic | linoleic | linolenic |
| BD | 25.6±1.1 | 1.51±0.1 | 11.2±0.0 | 59.1±1.3 | 2.40±0.30 |
| BDHCl | 25.6±1.2 | 1.71±0.2 | 10.6±0.7 | 60.0±0.9 | 1.92±0.24 |
| BDNaCl | 24.1±0.1 | 1.50±0.1 | 10.2±0.3 | 61.8±0.2 | 2.16±0.07 |
| CMHex | 9.61±0.4 | 1.64±0.2 | 14.7±0.6 | 71.0±0.2 | 2.97±0.21 |
| Hara | 22.9±0.5 | 1.34±0.1 | 11.3±0.3 | 61.9±0.9 | 2.46±0.10 |
| Methanol | 22.8±0.5 | 0.72±0.0 | 11.1±0.9 | 63.4±0.4 | 1.84±0.01 |
| Hexane | 22.1±0.3 | 0.90±0.1 | 12.2±0.3 | 62.3±0.2 | 2.38±0.27 |
|  |  |  |  |  |  |
|  | Maize starch | | | | |
|  | palmitic | stearic | oleic | linoleic | linolenic |
| BD | 42.2±1.4 | 2.56±0.44 | 2.56±0.44 | 52.5±3.7 | nd |
| BDHCl | 37.0±1.3 | 3.04±0.13 | 4.35±0.04 | 55.6±1.4 | nd |
| BDNaCl | 40.1±3.6 | 5.07±0.51 | 3.63±2.02 | 51.1±1.1 | nd |
| CMHex | 59.1±4.2 | 13.6±0.9 | 3.35±0.77 | 23.8±2.5 | nd |
| Hara | 39.7±0.2 | 21.1±0.5 | 17.8±0.70 | 21.2±0.9 | nd |
| Methanol | 27.2±1.6 | 1.62±0.24 | 9.04±1.13 | 62.0±2.9 | nd |
| Hexane | 42.1±1.5 | 19.7±0.5 | 14.0±0.0 | 24.0±1.0 | nd |
|  |  |  |  |  |  |
|  | Tapioca starch | | | | |
|  | palmitic | stearic | oleic | linoleic | linolenic |
| BD | 50.5±4.7 | 22.8±1.5 | 18.2±1.5 | 8.16±7.73 | nd |
| BDHCl | 45.7±0.7 | 25.0±0.4 | 21.0±1.4 | 8.20±1.14 | nd |
| BDNaCl | 44.4±0.1 | 22.2±0.3 | 22.1±0.2 | 11.1±0.2 | nd |
| CMHex | 50.0±0.0 | 30.0±0.6 | 15.0±0.2 | 5.01±0.31 | nd |
| Hara | 43.6±1.2 | 28.9±0.5 | 21.8±0.6 | 5.66±1.28 | nd |
| Methanol | 44.1±0.1 | nd | 44.3±3.4 | 11.5±3.3 | nd |
| Hexane | 65.6±0.4 | 34.3±0.4 | nd | nd | nd |
